# Selection-driven cost-efficiency optimization of transcripts modulates gene evolutionary rate in bacteria

**DOI:** 10.1186/s13059-018-1480-7

**Published:** 2018-07-31

**Authors:** Emily A. Seward, Steven Kelly

**Affiliations:** Department of Plant Sciences, University of Oxford, South Parks Road, Oxford, OX1 3RB UK

**Keywords:** Gene evolution, Synonymous codon use, Codon bias, Translational efficiency, Bacteria, Natural selection, Transcript optimization, Molecular evolution

## Abstract

**Background:**

Most amino acids are encoded by multiple synonymous codons. However, synonymous codons are not used equally, and this biased codon use varies between different organisms. It has previously been shown that both selection acting to increase codon translational efficiency and selection acting to decrease codon biosynthetic cost contribute to differences in codon bias. However, it is unknown how these two factors interact or how they affect molecular sequence evolution.

**Results:**

Through analysis of 1320 bacterial genomes, we show that bacterial genes are subject to multi-objective selection-driven optimization of codon use. Here, selection acts to simultaneously decrease transcript biosynthetic cost and increase transcript translational efficiency, with highly expressed genes under the greatest selection. This optimization is not simply a consequence of the more translationally efficient codons being less expensive to synthesize. Instead, we show that transfer RNA gene copy number alters the cost-efficiency trade-off of synonymous codons such that, for many species, selection acting on transcript biosynthetic cost and translational efficiency act in opposition. Finally, we show that genes highly optimized to reduce cost and increase efficiency show reduced rates of synonymous and non-synonymous mutation.

**Conclusions:**

This analysis provides a simple mechanistic explanation for variation in evolutionary rate between genes that depends on selection-driven cost-efficiency optimization of the transcript. These findings reveal how optimization of resource allocation to messenger RNA synthesis is a critical factor that determines both the evolution and composition of genes.

**Electronic supplementary material:**

The online version of this article (10.1186/s13059-018-1480-7) contains supplementary material, which is available to authorized users.

## Background

Production of proteins is a primary consumer of cell resources [1]. It requires allocation of cellular resources to production of RNA sequences as well as allocation of resources to production of nascent amino acid chains. Whilst a protein’s amino acid sequence is functionally constrained, redundancy in the genetic code means that multiple nucleotide sequences can code for the same protein. Since the biosynthetic cost and translational efficiency of synonymous codons vary, biased use of synonymous codons makes it possible to reduce the expenditure of cellular resources on messenger RNA (mRNA) production without altering the encoded protein sequence. Thus, it is possible to reduce resource allocation to protein synthesis without altering the encoded protein or affecting protein abundance. This is done by reducing transcript sequence cost or by increasing the efficiency with which those transcripts can be translated into protein. Consistent with this, it has been demonstrated that natural selection acts both to reduce the biosynthetic cost of RNA sequences [2, 3] and to increase the efficiency with which those RNA sequences can template the encoded polypeptide chain [4–10]. However, though selection has been shown to act on codon biosynthetic cost and translational efficiency independently, it is unknown how these two factors interact or whether optimization of one factor inherently results in optimization of the other. Note that, in addition to factors acting on resource allocation, functional constraints are also known to bias patterns of codon use. Some examples include RNA structural constraints to facilitate thermal adaptation and translational initiation [11–13], RNA sequence constraints to preserve splice sites [14] and translational constraints to ensure accurate protein folding [15–17]. However, since those factors primarily act on individual sites or sets of sites within genes and are independent of resource allocation, they were not considered further in this analysis.

Different bacteria employ a mix of three different strategies to decode synonymous codons [18]. These strategies make use of ‘wobble’ base pairing between the third base of the codon and the first base of the anticodon to facilitate translation of all 61 sense codons using a reduced set of transfer RNAs (tRNAs). These strategies can be broadly classified according to the tRNA genes that are absent [18]. Bacteria that utilize strategy 1 do not have tRNA genes harbouring an A residue at position 34 in the anticodon (A_34_NN), and therefore codons in transcripts with U in the third position (NNU_3_) are read by G_34_NN tRNAs via G:U base pairing. This is the most commonly used strategy and is employed by most bacteria for most four-codon boxes (excluding the four-codon box for arginine) [18, 19]. Strategy 2 is an extension of strategy 1 whereby tRNA genes harbouring a C residue at position 34 in the anticodon (C_34_NN) are absent, and therefore codons in transcripts with G in the third position (NNG_3_) are read by U_34_NN tRNAs via G:U base pairing. Similarly, strategy 3 is an extension of strategy 2 and involves reading all four synonymous codons (for four-codon boxes only) with a single U_34_NN tRNA.

As the translational efficiency of a codon is a function of the number of tRNAs that can translate that codon, and as different species encode different subsets of tRNA genes, the same codon is not necessarily equally translationally efficient in all species. Moreover, wobble pairing also influences the direction of translational selection such that it is different between different codon families [19]. In contrast, the biosynthetic cost of a codon of RNA is determined by the number and type of atoms contained within that codon and the number of high-energy phosphate bonds required for their assembly. The biosynthetic cost of a codon of RNA is independent of the biosynthetic cost of the amino acid it encodes, and thus variance in amino acid biosynthetic cost would not have a direct effect on the relative frequency of synonymous codon use. As the translational efficiency of a given codon varies between species but the biosynthetic cost of the codon remains the same, it was hypothesized that this must create a corresponding variation in the codon cost-efficiency trade-off between species. For example, biosynthetically cheap codons might be translationally efficient in one species but inefficient in another. We further hypothesized that variation in the codon cost-efficiency trade-off would limit the extent to which a transcript could be optimized to be both biosynthetically inexpensive and translationally efficient.

Here, we show that natural selection acts genome-wide to reduce cellular resource allocation to mRNA synthesis by solving the multi-objective optimization problem of minimizing transcript biosynthetic cost whilst simultaneously maximizing transcript translational efficiency. We show that this optimization is achieved irrespective of the codon cost-efficiency trade-off of a species, and that the extent to which resource allocation is optimized is a function of the production demand of that gene. Finally, we reveal that selection-driven optimization of resource allocation provides a novel mechanistic explanation for differences in evolutionary rates between genes and for the previously unexplained correlation in synonymous and non-synonymous mutation rates of genes.

## Results

### Selection acts to reduce biosynthetic cost and increase translational efficiency of transcript sequences

**Fig. 1:**
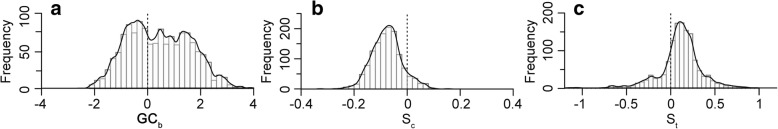
Bacterial genomes show selection to reduce nucleotide biosynthetic cost (*S*_c_) and increase translational efficiency (*S*_t_). Genome-wide values for 1320 bacterial species covering 730 genera for (**a**) bias towards GC (*GC*_b_). Positive values indicate bias towards GC. Negative values indicate bias towards AT. **b** Strength of selection acting on codon biosynthetic cost (*S*_c_). Negative values indicate selection acting to reduce biosynthetic cost. **c** Strength of selection acting on codon translational efficiency (*S*_t_). Positive values indicate selection acting to increase codon translational efficiency

Although selection has been shown to reduce resource allocation to mRNA production by reducing the biosynthetic cost of a codon of RNA or increasing translational efficiency independently [2–10], it is unknown how these two factors interact or whether optimization of one factor inherently results in optimization of the other. To address this, an analysis was conducted on 1320 bacterial species (Additional file 1) representing 730 different genera to establish if they were either under selection to increase codon translational efficiency, reduce codon biosynthetic cost or a combination of the two. For each species, genome-wide values for genome-wide GC bias (*GC*_b_), selection on transcript translational efficiency (*S*_t_) and selection on transcript biosynthetic cost (*S*_c_) were inferred (Fig. 1, Additional file 1). This was done using the complete set of open reading frames and tRNAs encoded in that species’ genome using the SK model [2] implemented using CodonMuSe (see Methods). Genome-wide GC content varied from 26 to 75% and so encompassed almost the entire range of known bacterial genome GC values [20]. This large variation in content was reflected in the range of values observed for *GC*_b_ (Fig. 1a, mean = 0.44). Of the 1320 species in this analysis, 91% had negative *S*_c_ values (mean *S*_c_ = − 0.08), indicating a genome-wide selective pressure to reduce the biosynthetic cost of transcript sequences through biased synonymous codon use (Fig. 1b). This observation is consistent with previous studies that revealed analogous effects when nitrogen or energy was limited [2, 3]. Similarly, 78% of species had positive values for *S*_t_ (mean *S*_t_ = 0.1), indicating a genome-wide selective pressure to increase the translational efficiency of transcript sequences (Fig. 1c). This is consistent with multiple examples where a strong pressure has been shown to favour high translational efficiency [4–10]. Moreover, 74% of species had both a negative *S*_c_ value and a positive *S*_t_ value, demonstrating that selection is not mutually exclusive when acting on translational efficiency and codon biosynthetic cost. Indeed, the majority of species experience selection to reduce transcript biosynthetic cost whilst simultaneously maximizing transcript translational efficiency.

### More translationally efficient bacterial codons are generally more biosynthetically costly

**Fig 2:**
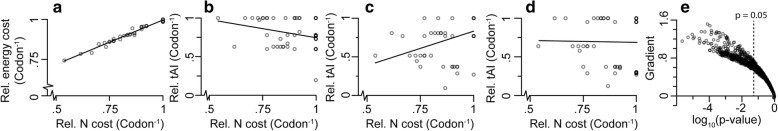
Different tRNA sparing strategies alter a species’ codon cost-efficiency trade-off. **a** Codon nitrogen cost (N cost) correlates almost perfectly with codon energetic cost (*p* < 0.05, *y* = 0.6*x* + 0.44, *R*^2^ = 0.98). **b** A full complement of tRNAs has a negative correlation between codon biosynthetic cost and translational efficiency (tAI) (*p* < 0.05, *y* = − 0.5*x* + 1.21, *R*^2^ = 0.10). **c** tRNA sparing strategy 1 (NNU codons translated by GNN anticodons) has a positive correlation between codon biosynthetic cost and translational efficiency (*p* < 0.05, *y* = 0.9*x* – 0.06, *R*^2^ = 0.18). **d** tRNA sparing strategy 2 (strategy 1 + NNG codons translated by UNN anticodons) has no significant correlation between codon biosynthetic cost and translational efficiency (*p* > 0.05, *y* = 0.74, *R*^2^ = 0). **e** None of the 1320 bacterial species in this analysis have a significant negative correlation between codon cost and translational efficiency (*p* > 0.05). The *y*-axis is the gradient of the line of best fit between codon biosynthetic cost and translational efficiency

The biosynthetic cost of a codon can be defined as the number and type of atoms contained within the codon or the number of high-energy phosphate bonds required for their assembly. Natural selection acting on biosynthetic cost, both in terms of nitrogen atoms [2] or energetic requirements [3], has been shown to play a role in promoting biased patterns of synonymous codon use. However, as the energy and nitrogen costs of a codon correlate almost perfectly (Fig. 2a), it is not possible to distinguish which factor is responsible for biased patterns of codon use in the absence of additional information about the biology of the organism in question. Nonetheless, given the near-perfect correlation, analysis of selection acting on overall codon biosynthetic cost can be approximated by analysis of either nitrogen or energetic requirements.

Codon translational efficiency is generally measured using the tRNA adaptation index (tAI), which considers both the abundance of iso-accepting tRNAs and wobble base pairing [21]. Since tRNA gene copy number varies between species, there is a corresponding variation in the relative translational efficiency of their associated codons [18, 22]. Therefore, the relationship between codon biosynthetic cost and codon translational efficiency (referred to from here on as the codon cost-efficiency trade-off) must vary between species. For example, a hypothetical species encoding a full complement of Watson-Crick pairing tRNA genes (i.e. 61 tRNA genes for 61 codons), each present as a single copy, would have a weak negative correlation between codon biosynthetic cost and codon translational efficiency (Fig. 2b). In contrast, a hypothetical species that employed tRNA sparing strategy 1 (no ANN tRNAs) or strategy 2 (no ANN or CNN tRNAs) [18] would show a positive (Fig. 2c) or no (Fig. 2d) correlation between cost and efficiency respectively. Therefore, a broad range of codon cost-efficiency trade-offs is possible, and the magnitude/gradient of this trade-off is dependent on the tRNA gene copy number of a given species.

None of the 1320 species used in this analysis contained a full complement of tRNAs. Moreover, only two species strictly adhered to a single sparing strategy for all synonymous codon groups (e.g. *Escherichia coli* uses strategy 2 for decoding alanine but strategy 1 for decoding glycine). Given that neither tRNA sparing strategy 1 nor 2 led to a negative correlation between cost and efficiency, it is therefore expected that species would have either a positive or no correlation between codon cost and efficiency. Furthermore, given the many different potential tRNA complements, it is anticipated that a continuum of gradients in trade-off between cost and efficiency would be observed. To assess this, the codon cost-efficiency trade-off was calculated for the 1320 bacterial species (Fig. 2e). As expected, species with a significant negative correlation between cost and efficiency were not observed. Instead, all species exhibited either positive or non-significant correlations between codon cost and efficiency (Fig. 2e). Thus in general, the synonymous codons that are most translationally efficient are those that consume the most resources for biosynthesis.

**Fig. 3:**
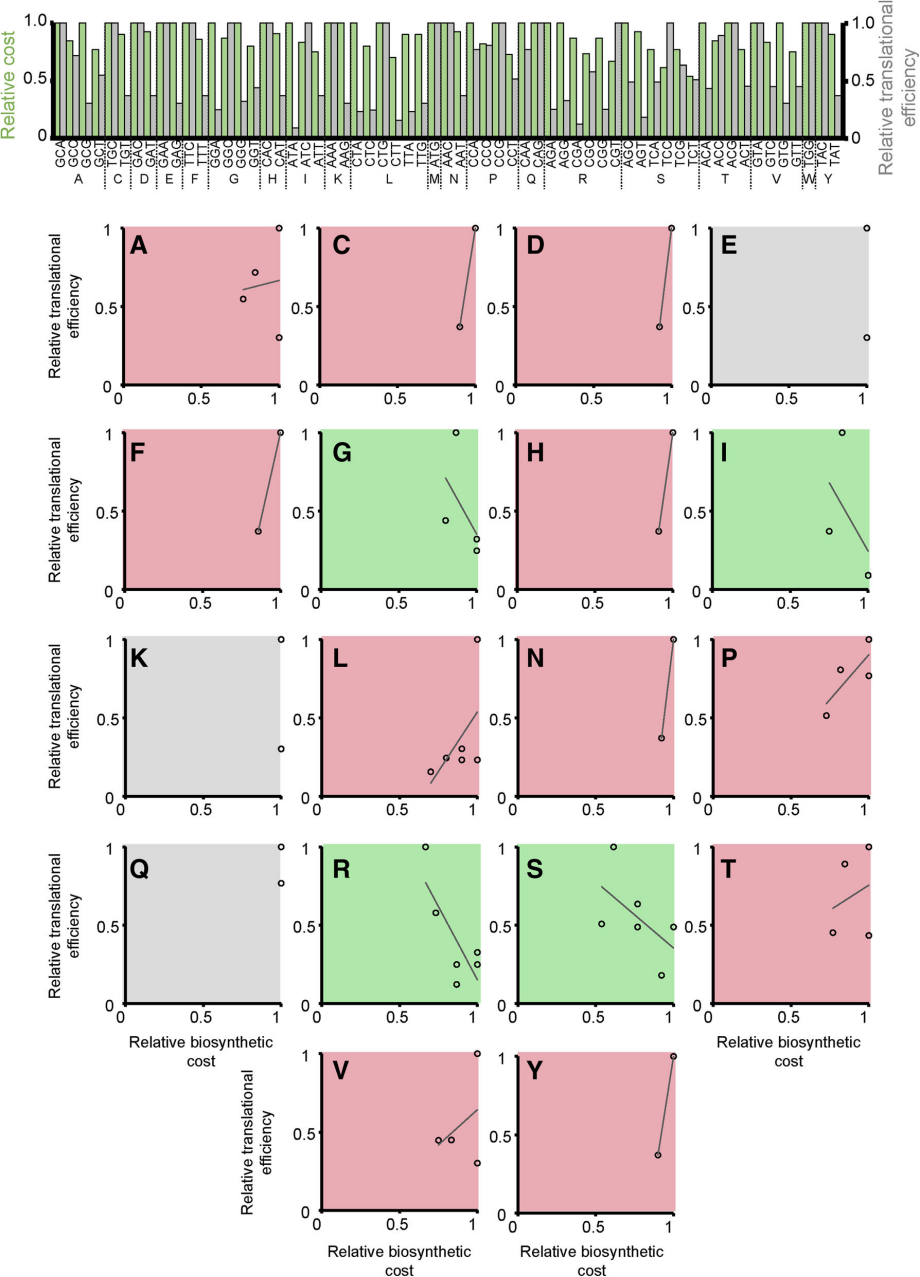
The relationship between codon biosynthetic cost and codon translational efficiency in *E. coli*. The *top panel* contains a bar chart that shows the relative biosynthetic cost (*green bars*) and relative translational efficiency (*grey bars*) of each synonymous codon for each amino acid. The *bottom panel* contains scatter plots of this cost-efficiency data for each amino acid with multiple synonymous codons. The shading of the plots indicates the slope of the fitted line between relative biosynthetic cost and relative translational efficiency. *Red shading* positive correlation, *green shading* negative correlation, *grey shading* no correlation. *A* alanine, *C* cysteine, *D* aspartic acid, *E* glutamic acid, *F* phenylalanine, *G* glycine, *H* histidine, *I* isoleucine, *K* lysine, *L* leucine, *N* asparagine, *P* proline, *Q* glutamine, *R* arginine, *S* serine, *T* threonine, *V* valine, *Y* tyrosine

Note that the only way to avoid a cost-efficiency trade-off is if there is a perfect negative correlation (*R*^2^ = 1.0) between codon biosynthetic cost and codon translational efficiency such that any reduction in cost is mirrored by an increase in translational efficiency. For example*, E. coli* does not have a significant positive (or negative) relationship between codon cost and codon translational efficiency (*R*^2^ = 0.02). This means that sometimes the more expensive codons are more translationally efficient and sometimes they are less translationally efficient; there is no overall trend (Fig. 3). For example, the codon GCA encoding alanine is both the most expensive codon for alanine and the most translationally efficient codon for alanine (Fig. 3). Thus, when selection acts to simultaneously reduce cost and increase translational efficiency, these two selective forces are acting in opposition on codon GCA for alanine (i.e. *S*_c_ disfavours GCA whilst *S*_t_ favours GCA). Thus, there is a cost-efficiency trade-off for the synonymous codons encoding alanine. This phenomenon, where the two selective forces act in opposition, is observed for 11 of the 18 amino acids with multiple synonymous codons in *E. coli* (Fig. 3, red shaded plots). Thus, there is a cost-efficiency trade-off for 11 of the 18 amino acids in *E. coli*. For three of the codons there is no trade-off because, although the two available synonymous codons have different translational efficiencies, they have the same biosynthetic cost (Fig. 3, grey shaded plots). For the remaining four amino acids there is a negative correlation between codon biosynthetic cost and codon translational efficiency (Fig. 3, green shaded plots). However, even for these codons with a negative relationship between cost and efficiency it is not guaranteed that when mutating from one codon to another that both biosynthetic cost and translational efficiency will change in the same direction (Fig. 3, green shaded plots). For example, even though the codons for isoleucine exhibit a negative correlation (Fig. 3I), the cheapest codon for isoleucine is the least translationally efficient and thus *S*_t_ and *S*_c_ act in opposition on this codon. Thus, for any organism that does not have a perfect negative correlation between biosynthetic cost and translational efficiency there is an inherent cost-efficiency trade-off. Moreover, as the relationship between biosynthesis cost and translational efficiency is different for each synonymous codon group, *S*_c_ and *S*_t_ thus act antagonistically or synergistically on each codon such that the direction of selection is different for each synonymous codon group. This provides a novel mechanism that helps to explain the differences in the direction of selection that have been observed between different codon groups [23].

### Genes that experience the strongest selection for increased transcript translational efficiency are also under the strongest selection to reduce biosynthetic cost

**Fig. 4:**
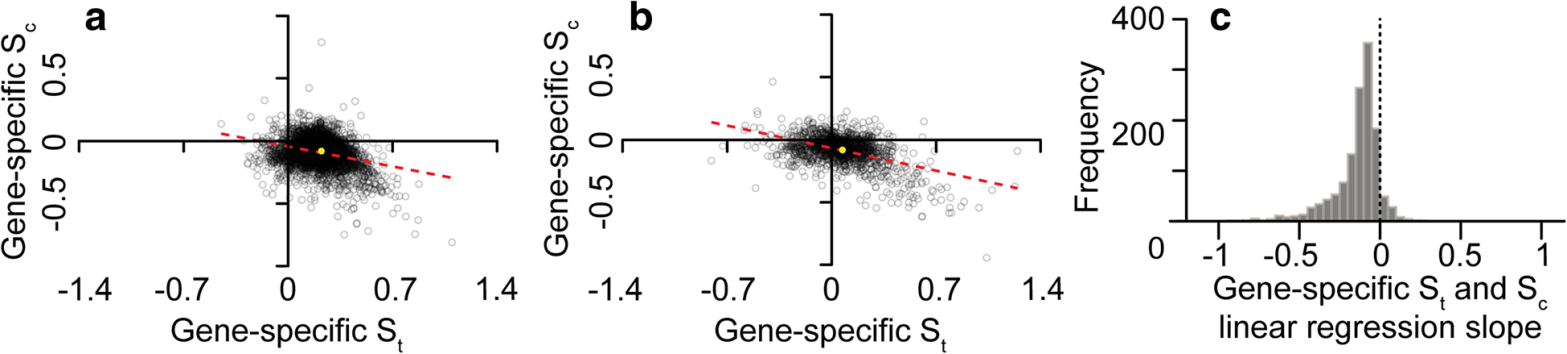
The genes under the strongest selection for translational efficiency (*S*_t_) are also under the strongest selection to reduce biosynthetic cost (*S*_c_). Scatter plots of gene-specific *S*_t_ and *S*_c_ values for (**a**) *Escherichia coli*, (**b**) *Lactobacillus amylophilus*. In both cases the line of best fit is shown (*red*), and the *yellow dot* is the genome-wide best-fit value for each species. Each point has been set to an opacity of 20% so density can be judged. **c** Histogram of the slope between *S*_c_ and *S*_t_ for individual genes for each of the 1320 bacterial species in this analysis

Given that the majority of species exhibited selection to reduce cost and increase translational efficiency at the genome-wide level (irrespective of the magnitude of their cost-efficiency trade-off), the extent to which this was also seen at the level of an individual gene within species was determined. Here, the strength of selection acting on transcript translational efficiency and the strength of selection on transcript biosynthetic cost were inferred for each individual gene in each species. The relationship between *S*_c_ and *S*_t_ was then compared for each species. For example, in *E. coli*, which does not have a strong cost-efficiency trade-off, there is a significant negative correlation between *S*_c_ and *S*_t_ (Fig. 4a). Here, the genes that experienced the greatest selection to increase efficiency are those that experienced the greatest selection to reduce biosynthetic cost. The same phenomenon was also observed for *Lactobacillus amylophilus*, a species with a strong codon cost-efficiency trade-off (Fig. 4b). Overall, significant correlations between *S*_c_ and *S*_t_ for individual genes were observed for 91% of species (*p* < 0.05, Fig. 4c). Therefore, irrespective of the magnitude of the codon cost-efficiency trade-off, selection is performing multi-objective optimization of transcript sequences to reduce their biosynthetic cost whilst increasing their translational efficiency and thereby reducing resource allocation to mRNA production.

**Fig. 5:**
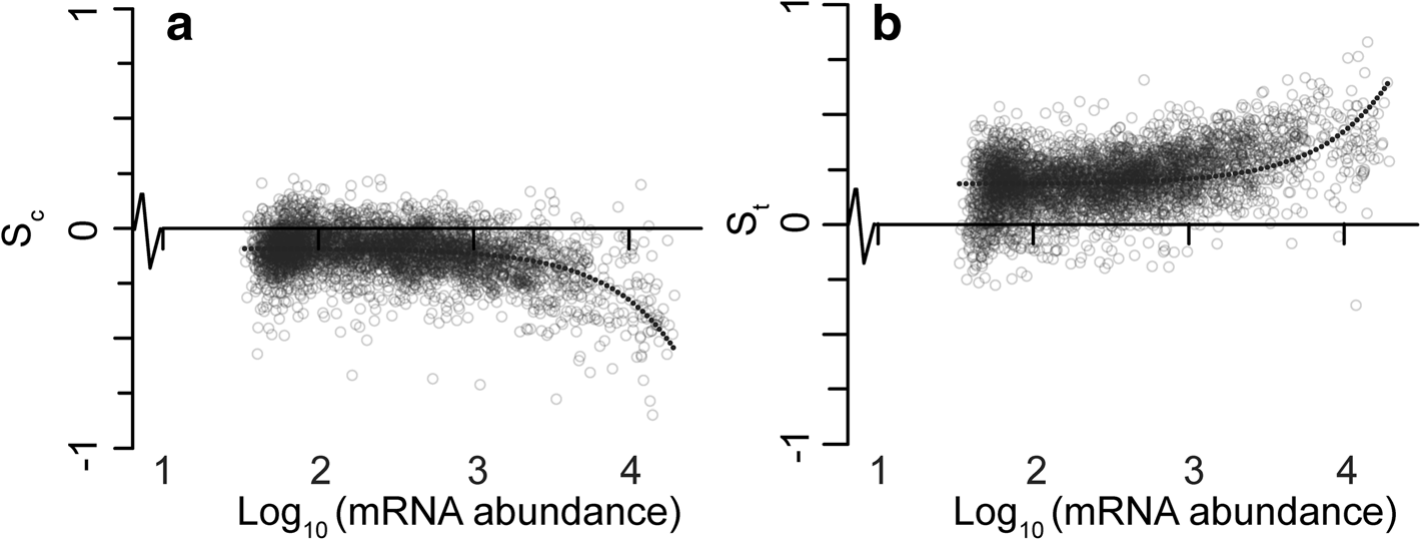
Selection acts in proportion to mRNA abundance to decrease codon biosynthetic cost and increase codon translational efficiency in *Escherichia coli*. **a** There is a negative correlation between selection acting on codon biosynthetic cost (*S*_c_) and mRNA abundance. The linear line of best fit (shown here on a log scale) has an *R*^2^ value of 0.18. **b** There is a positive correlation between selection acting to increase codon translational efficiency (*S*_t_) and gene expression. The linear line of best fit (shown here on a log scale) has an *R*^2^ value of 0.13. Each point has been set to an opacity of 20% so density can be judged

As the most highly expressed genes in a cell comprise the largest proportion of cellular RNA, the strength of selection experienced by a gene is thought to be dependent on the mRNA abundance of that gene [24–26]. In agreement with this, evaluation of the relative mRNA abundance of genes in *E. coli* revealed that the most highly expressed genes exhibited the greatest selection to reduce transcript biosynthetic cost (Fig. 5a) whilst also showing the strongest selection to increase transcript translational efficiency (Fig. 5b). Thus, selection acts in proportion to relative mRNA abundance to perform multi-objective optimization of codon bias in order to reduce resource allocation to transcript sequences through production of low-cost, high-efficiency transcripts.

### Sequence optimization for cost and efficiency constrains molecular evolutionary rate

Given that codon choice has been shown to provide a selective advantage per codon per generation [27], it was hypothesized that the extent to which a transcript is jointly optimized for codon cost and efficiency would constrain the rate at which the underlying gene sequence can evolve. Specifically, the more highly optimized a transcript is for both biosynthetic cost and translational efficiency, the higher the proportion of spontaneous mutations that would reduce the cost-efficiency optimality of the transcript sequence. Therefore, spontaneous mutations in highly optimized genes are more likely to be deleterious than spontaneous mutations in less optimized genes. As deleterious mutations are lost more rapidly from the population than neutral mutations, the more highly optimized a gene sequence is, the lower its apparent evolutionary rate should be.

**Fig. 6:**
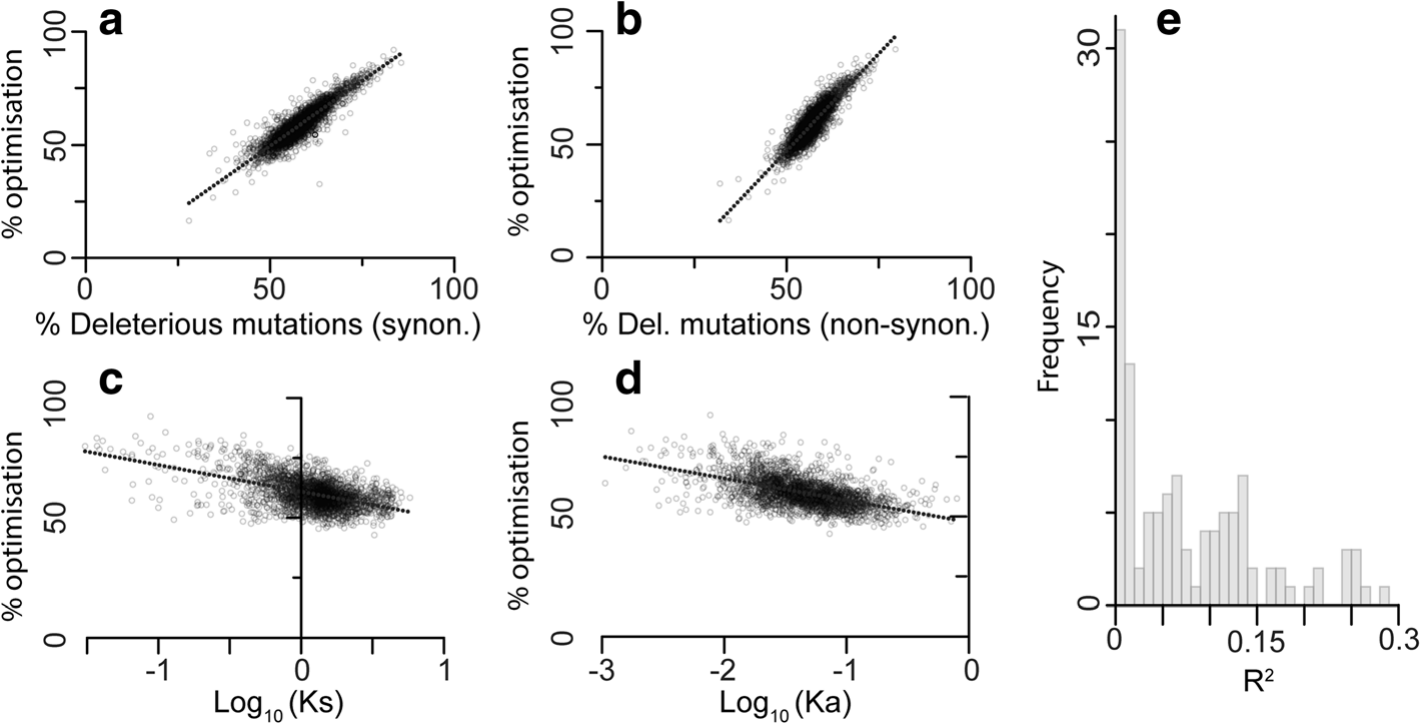
Selection-driven optimization of resource allocation is a critical factor that determines molecular evolutionary rate. Highly cost-efficiency optimized genes have a higher proportion of deleterious (**a**) synonymous (*y* = 1.15*x* – 8, *R*^2^ = 0.81) and (**b**) non-synonymous (*y* = 1.71*x* − 38, *R*^2^ = 0.78) mutations. Orthologous genes in *Escherichia coli* and *Salmonella enterica* show a negative correlation between sequence cost-efficiency optimization and the rate of (**c**) synonymous mutations (*K*_s_) (*y* = − 11*x* + 61, *R*^2^ = 0.26) and (**d**) non-synonymous mutations (*K*_a_) (*y* = − 9*x* + 48, *R*^2^ = 0.28). **e** Histogram of proportion of gene evolutionary rate explained by selection-driven cost-efficiency optimization of transcript sequences

To test this hypothesis, the complete set of gene sequences from *E. coli* was subject to stochastic in silico mutagenesis, and the proportion of single nucleotide mutations that resulted in reduced transcript cost-efficiency optimality was evaluated. As expected, the proportion of deleterious mutations increased linearly with transcript sequence optimality. This effect was seen for both synonymous (Fig. 6a) and non-synonymous mutations (Fig. 6b). The effect in non-synonymous mutations is seen because a single base mutation from an optimal codon encoding one amino acid is unlikely to arrive at an equally optimal (or better) codon encoding any other amino acid. Thus as expected, the more optimal a codon is, the less likely a spontaneous mutation will result in a codon with higher optimality irrespective of whether that codon encodes the same amino acid.

The extent to which transcript sequences in *E. coli* were jointly cost-efficiency optimized was compared to the synonymous (*K*_s_) and non-synonymous (*K*_a_*)* mutation rate of that gene, estimated from comparison with *Salmonella enterica*. Consistent with the hypothesis, the rates of synonymous (*K*_s_, Fig. 6c) and non-synonymous (*K*_a_, Fig. 6d) changes were directly proportional to the extent to which the gene sequence had been optimized by natural selection for low biosynthetic cost and high translational efficiency (Fig. 6a and b). Joint optimization of biosynthetic cost and translational efficiency can explain 26% and 28% of variation in synonymous and non-synonymous gene evolutionary rate (Fig. 6c and d). Whilst efficiency optimization explained more of the variance in gene evolutionary rate, the linear regression model that considered both cost and efficiency optimization was significantly better than models that considered either factor alone, whether or not derived optimization values or raw tAI and biosynthetic costs were considered (Additional file 2: Figure S1, analysis of variance (ANOVA), *p* < 0.001). Therefore, this analysis provides a mechanistic explanation for previous studies that found a strong correlation between non-synonymous evolutionary rate and mRNA abundance [24]. Moreover, it explains more variation in gene evolutionary rate for the same species than previous studies that focused on the deleterious effects of protein mis-folding [17, 24].

To determine if this relationship was also observed for other bacteria, an additional 176 species pairs were analysed (Fig. 6e, Additional file 3). Of these species pairs, 81% were consistent with the observation for *E. coli* and *S. enterica*, such that variance in selection-driven gene sequence optimization explained on average 6.9% of variance in *K*_s_ between genes (Fig. 6e, Additional file 3). Also consistent with the analysis for *E. coli* and *S. enterica*, the linear regression model that considered joint cost-efficiency optimization explained more variance in gene evolutionary rate than either cost optimization (4.4%) or translation optimization (5.9%) alone. Thus, the extent to which transcript sequences are jointly optimized for biosynthetic cost and translational efficiency can explain a significant component of variation in gene evolutionary rate. Moreover, selection-driven cost-efficiency optimality is also sufficient to explain the correlation between the rates of synonymous and non-synonymous mutations.

## Discussion

Differences in molecular evolutionary rates between species are thought to be mainly due to differences in organism generation time [28]. However, differences in evolutionary rates between genes in the same species lack a complete mechanistic explanation. Prior to the study presented here, it was known that functional constraints of the encoded protein sequence contribute to the constraint of the rate of non-synonymous changes [29]. It had also been observed that mRNA abundance and patterns of codon bias correlated with the evolutionary rate of genes [30, 31] and that rates of synonymous and non-synonymous changes were correlated [32]. The study presented here unifies these prior observations and provides a mechanistic explanation for both variation and correlation in molecular evolutionary rates of genes. Specifically, this study shows that stochastic mutations in gene sequences are more likely to result in deleterious alleles in proportion to the extent to which that gene sequence has been jointly optimized by natural selection for reduced transcript biosynthetic cost and enhanced translational efficiency.

**Fig. 7:**
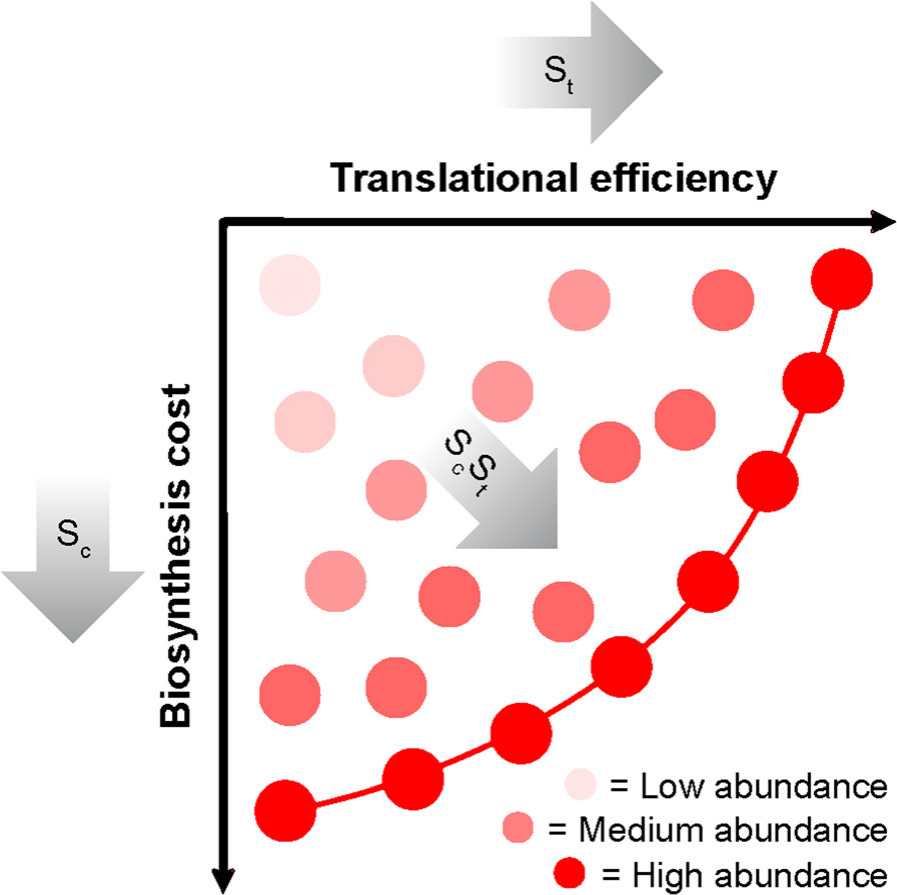
A cartoon depicting how selection optimizes both biosynthetic cost and translational efficiency for individual genes. Each *circle* represents a hypothetical gene sequence. The *intensity of the color* of the circle is proportional to its mRNA abundance. Low abundance genes experience weak selection and are not optimized for biosynthetic cost or translational efficiency. Highly expressed genes experience strong selection and lie on or near to the Pareto efficient frontier (indicated by a *red line*). The *shaded arrows* show the direction that selection will move a gene through this landscape. It is not possible for a gene to occur in the space beyond the Pareto frontier because of the cost-efficiency trade-off

The mechanism provided here also explains the relationship between mRNA abundance and gene evolutionary rate. Specifically, functional constraints on protein abundance stipulate the quantity of mRNA required to produce that protein. The more mRNA that is required, the greater the percentage of total cellular resources that must be invested within the transcript. The mechanism simply entails that the more transcript that is present, the stronger the selective pressure will be to reduce the cellular resources committed to that transcript. Importantly, minimizing these resources can be achieved both by using codons that require fewer resources for their biosynthesis or by utilizing translationally efficient codons that increase the protein-to-transcript ratio and therefore reduce the amount of transcript required to produce the same amount of protein (Fig. 7). Overall, this study reveals how the economics of gene production is a critical factor in determining both the evolution and composition of genes.

## Conclusions

Codon use is biased across the tree of life, with patterns of bias varying both between species and between genes within the same species. Here we demonstrate that variation in tRNA content between species creates a corresponding variation in the codon cost-efficiency trade-off whereby codons that cost the least to biosynthesize are not equally translationally efficient in all species. We show that, irrespective of the strength of the codon cost-efficiency trade-off, natural selection performs multi-objective gene sequence optimization so that transcript sequences are optimized to be both low cost and highly translationally efficient, and that the nature of this trade-off constrains the extent of the solution. We demonstrate that this multi-objective optimization is dependent on mRNA abundance, such that the transcripts that comprise the largest proportion of cellular mRNA are those that experience the strongest selection to be both low cost and highly efficient. Finally, we show that the extent to which a gene sequence is jointly optimized for reduced transcript cost and enhanced translational efficiency is sufficient to explain a significant proportion of the variation in the rate of gene sequence evolution. Furthermore, it is also sufficient to explain the phenomenon that the rate of synonymous and non-synonymous mutation for a gene is correlated [32].

## Methods

### Data sources

We obtained 1320 bacterial genomes from the National Center for Biotechnology Information (NCBI) (www.ncbi.nlm.nih.gov). In order to avoid over-sampling of more frequently sequenced genera, the number of species from each genus was restricted to 5, with a maximum of 1 strain (or subspecies or serovar) per species. Therefore, the 1320 species sampled in this study were distributed among 730 different genera. Only genes that were longer than 30 nucleotides, had no in-frame stop codons and began and ended with start and stop codons respectively were analysed. Each species in this analysis contained a minimum of 500 genes that fit these criteria. Full details of species names, genome accession numbers, strain details and selection coefficients are provided in Additional file 1.

### Evaluation of translational efficiency (tAI)

To obtain the number of tRNA genes in each genome, tRNAscan was run on each of the 1320 bacterial genomes [33]. This current version (1.4) of tRNAscan is unable to distinguish between tRNA-Met and tRNA-Ile with the anticodon CAT. Thus tRNA-Ile(CAT), whilst present, is not detected in any of the genomes. To compensate for this, a single copy of tRNA-Ile with the anticodon CAT was added to the tRNA counts for each species if more than one tRNA-Met(CAT) was found. The tRNA adaptation index (tAI) [22], which considers both the tRNA gene copy number and wobble base pairing when calculating the translational efficiency of a codon, was evaluated using the optimized *s*_*ij*_ values for bacteria obtained by Tuller et al. [34] and the equation developed by dos Reis et al. [21]. *s*_*uu*_ was set to 0.7 as proposed by Navon et al. [35], and *s*_*uc*_ was set to 0.95 as U_34_ has been shown to have weak codon-anticodon coupling with cytosine [36]. Each species in this analysis was able to translate all codons, was not missing key tRNAs and did not require unusual tRNA modifications.

The analysis presented here utilizes the same tAI constants described above for all species. Thus, the analysis does not account for anticodon base modifications that are species-specific. If present, such a modification will cause the tAI constant for a given codon to be incorrect in that species and may have an impact on the estimate of *S*_t_ for that species. However, the impact of this difference in tAI constant on the estimation of *S*_t_ will be proportional to the number of anticodons that are modified (out of the total pool of anticodons) and the magnitude of difference in tAI constant. This is a limitation of this large-scale multi-species analysis that is likely to have introduced some stochastic error into the results.

### Calculation of relative codon biosynthetic cost and translational efficiency

Codon biosynthetic cost and translational efficiency were calculated relative to other synonymous codons such that the synonymous codon with the greatest value had a relative cost or efficiency of 1. For example, the nitrogen cost of GCC is 11 atoms. The most expensive synonymous codon is GCG/GCA (13 atoms). Therefore, the relative biosynthetic cost of GCC is 11/13 = 0.85. The same evaluation was done to calculate codon translational efficiency.

We note that 4 of the 1320 species in this analysis do not encode a full complement of enzymes necessary to biosynthesize A, C, G, T and U nucleotides from phosphoribosyl pyrophosphate. These species comprise *Ureaplasma parvum*, *Ureaplasma urealyticum*, *Mycoplasma parvum* and *Mycoplasma pneumoniae*, and they depend on import of nucleotide precursors from their host [37]. These species also lack nucleoside diphosphate kinase (for converting deoxyribonucleoside diphosphates, (d)NDPs, to deoxyribonucleoside triphosphates, (d)NTPs), [38], and thus they catalyze these reactions using other enzymes [39]. Removal of these four species from this analysis does not affect the results presented in this work.

### CodonMuSe: a fast and efficient algorithm for evaluating drivers of codon usage bias

The SK model [2] was used to infer the joint contribution of genome-wide GC bias, selection acting on codon biosynthetic cost and selection acting on codon translational efficiency to biased synonymous codon use. We note here that the *GC*_b_ parameter in this study is a composite parameter that integrates into a single variable the multiple factors contributing to genome-wide GC content bias. Such factors include gene conversion [40], differences in repair efficiency [41], mutational biases during DNA replication [42–44] and selection acting on genome-wide GC content [45].

To facilitate the large-scale comparative application of this model, a rapid, stand-alone version was implemented in Python. The algorithm, instructions for use and example files are available for download at https://github.com/easeward/CodonMuSe. Further details about the algorithm can be found in Additional file 2.

For each species, the complete set of protein coding genes and the tRNA copy number inferred using tRNAscan were provided as input and CodonMuSe was run in default mode such that the optimal model selection was conducted automatically. For 1247 of the 1320 species (94.4%) the optimal model was determined to be the three-parameter model containing *GC*_b_, *S*_c_ and *S*_t_ (Additional file 1). To facilitate plotting parameter values for all species, the remaining 5.6% of species were run with a three-parameter model. In all cases the inferred value for the missing parameters was ~ 0 (Additional file 1). For example, for those species where *S*_t_ was not in the optimal model, the mean *S*_t_ value was 0.001, the mean value for species where *S*_t_ was included in the optimal model was 0.111.

### Comparing selection acting on codon bias and transcript abundance levels

Transcriptome data for *E. coli* str. K-12 MG1655 were downloaded from the NCBI (series GSE15534). The raw data were subjected to quantile normalization and background correction as implemented in the NimbleScan software package, version 2.4.27 [46, 47]. The three biological replicates for the logarithmic growth phase were available; however, the third replicate was inconsistent with the first two and so was excluded from this analysis. As each gene had multiple probes, the average probe value for each gene was taken. The three-parameter CodonMuSe model using the value for *GC*_b_ estimated from a genome-wide analysis was run for each of the 4099 genes in *E. coli* individually, and thus values for *S*_c_ and *S*_t_ were obtained for each gene. The values for these selection coefficients were plotted against the relative mRNA abundance data described above [46].

### Calculating the extent to which gene sequences were jointly optimized for biosynthetic cost and translational efficiency

To define the extent to which a sequence has been jointly optimized for both biosynthetic cost and translational efficiency, the relative Pareto optimality of each gene was calculated. To do this, the boundaries of sequence space were defined as in Additional file 2: Figure S2. Here, the cost-efficiency Pareto frontier is the full set of coding sequences that are Pareto efficient, where it is impossible to change the codons of the sequence to make the transcript cheaper without making it less efficient (or vice versa) (red frontier, Additional file 2: Figure S2). The opposite frontier is the full set sequences where it is impossible to change the codons of the sequence to make the transcript more expensive without making it more efficient (or vice versa) (blue frontier, Additional file 2: Figure S2). Thus, the extent to which transcript sequences were jointly optimized for both biosynthetic cost and translational efficiency was evaluated as the relative distance of a given gene to the cost-efficiency Pareto frontier for the sequence constrained by the amino acid sequence, i.e. $\left(\frac{d4}{d1+d4}\right)\ast 100$ (Additional file 2: Figure S2). Therefore, a value of 100% optimization represents a gene that lies on the Pareto frontier. Genes that are less than 100% optimized occupy the space between the cost-efficiency Pareto frontier (red frontier) and the opposite frontier (blue frontier, minimizing transcript efficiency or maximizing cost) for that amino acid sequence (Additional file 2: Figure S2).

### Calculation of molecular evolutionary rates

Molecular evolutionary rates (*K*_a_ and *K*_s_ values) were calculated for orthologous genes in *E. coli* and *S. enterica*. In total, 2468 single-copy orthologous genes were identified for *E. coli* and *S. enterica* using OrthoFinder version 1.1.4 [48]. These sequences were aligned at the amino acid level using MergeAlign [49], and this alignment was then rethreaded with the coding sequences to create codon-level nucleotide alignments. Only aligned sequences longer than 30 nucleotides with less than 10% gaps were used. Gapped regions were removed and KaKs_Calculator 2.0 [50] was run using the GMYN model to evaluate *K*_a_ and *K*_s_ values for each pair of aligned nucleotide sequences. As the molecular evolutionary rates represent the average of the mutation rates of the gene pair since they last shared a common ancestor, these rates were compared to the average optimality of the same gene pair in both species.

The same analysis was conducted on 1066 additional pairs of species obtained by exhaustive pairwise comparison of all species that were within the same genus. These 1066 pairwise comparisons were filtered to remove those with *K*_s_ saturation (i.e. mean *K*_s_ > 1) and fewer than 1000 genes. This filtered set contained 176 species pairs (Additional file 3).

### Linear regression analyses

All linear regression analyses were conducted using the lm package in R. In all cases, *p* values quoted are the *p* values for the linear regression model.

## Additional files

Additional file 1: NCBI accession numbers for the 1320 species used in this analysis. Also provided are the *R*^2^ values for the model fit between the real codon use frequencies and model fitted codon use frequencies and the fitted values for each model parameter. (XLSX 162 kb)

Additional file 2: **Figure S1**, **Figure S2** and the CodonMuSe algorithm. (PDF 964 kb)

Additional file 3: The *R*^2^ and *p* values for the fit between *K*_s_ and cost-efficiency optimization for the 176 species pairs with mean *K*_s_ < 1 and more than 1000 genes. Mean *K*_s_, mean cost-efficiency optimization, *R*^2^ and *p* value of fit are provided. (XLSX 25 kb)

## References

[1] Farmer IS, Jones CW (1976). The energetics of Escherichia coli during aerobic growth in continuous culture. Eur J Biochem.

[2] Seward EA, Kelly S (2016). Dietary nitrogen alters codon bias and genome composition in parasitic microorganisms. Genome Biol.

[3] Chen W-H, Lu G, Bork P, Hu S, Lercher M (2016). Energy efficiency trade-offs drive nucleotide usage in transcribed regions. Nat comminications.

[4] Horn D (2008). Codon usage suggests that translational selection has a major impact on protein expression in trypanosomatids. BMC Genomics.

[5] Rocha EPC (2004). Codon usage bias from tRNA’s point of view: redundancy, specialization, and efficient decoding for translation optimization. Genome Res.

[6] Sørensen MA, Kurland CG, Pedersen S (1989). Codon usage determines translation rate in Escherichia coli. J Mol Biol.

[7] Hu H, Gao J, He J, Yu B, Zheng P, Huang Z (2013). Codon optimization significantly improves the expression level of a keratinase gene in Pichia pastoris. PLoS One.

[8] Akashi H (1994). Synonymous codon usage in Drosophila melanogaster: natural selection and translational accuracy. Genetics.

[9] Shah P, MA G (2011). Explaining complex codon usage patterns with selection for translational efficiency, mutation bias, and genetic drift. Proc Natl Acad Sci U S A.

[10] Precup J, Parker J (1987). Missense misreading of asparagine codons as a function of codon identity and context. J Biol Chem.

[11] Lao PJ, Forsdyke DR (2000). Thermophilic bacteria strictly obey Szybalski’s transcription direction rule and politely purine-load RNAs with both adenine and guanine. Genome Res.

[12] Paz A, Mester D, Baca I, Nevo E, Korol A (2004). Adaptive role of increased frequency of polypurine tracts in mRNA sequences of thermophilic prokaryotes. Proc Natl Acad Sci U S A.

[13] Goodman DB, Church GM, Kosuri S (2013). Causes and effects of N-terminal codon bias in bacterial genes. Science.

[14] Eskesen ST, Eskesen FN, Ruvinsky A (2004). Natural selection affects frequencies of AG and GT dinucleotides at the 5′ and 3′ ends of exons. Genetics.

[15] Novoa EM, Ribas de Pouplana L (2012). Speeding with control: codon usage, tRNAs, and ribosomes. Trends Genet.

[16] Zhang F, Saha S, Shabalina SA, Kashina A (2010). Differential arginylation of actin isoforms is regulated by coding sequence-dependent degradation. Science.

[17] Drummond DA, Wilke CO (2008). Mistranslation-induced protein misfolding as a dominant constraint on coding-sequence evolution. Cell.

[18] Grosjean H, de Crécy-Lagard V, Marck C (2010). Deciphering synonymous codons in the three domains of life: co-evolution with specific tRNA modification enzymes. FEBS Lett.

[19] Ran W, Higgs PG (2010). The influence of anticodon-codon interactions and modified bases on codon usage bias in bacteria. Mol Biol Evol.

[20] Brocchieri L (2013). The GC content of bacterial genomes. Phylogenetics Evol Biol.

[21] dos Reis M, Savva R, Wernisch L (2004). Solving the riddle of codon usage preferences: a test for translational selection. Nucleic Acids Res.

[22] dos Reis M, Wernisch L, Savva R (2003). Unexpected correlations between gene expression and codon usage bias from microarray data for the whole Escherichia coli K-12 genome. Nucleic Acids Res.

[23] Higgs PG, Ran W (2008). Coevolution of codon usage and tRNA genes leads to alternative stable states of biased codon usage. Mol Biol Evol.

[24] Drummond DA, Wilke CO (2009). The evolutionary consequences of erroneous protein synthesis. Nat Rev Genet.

[25] Ran W, Higgs PG (2012). Contributions of speed and accuracy to translational selection in bacteria. PLoS One.

[26] Pal C, Papp B, Hurst LD (2001). Highly expressed genes in yeast evolve slowly. Genetics.

[27] Brandis G, Hughes D (2016). The selective advantage of synonymous codon usage bias in Salmonella. PLoS Genet.

[28] Weller C, Wu M (2015). A generation-time effect on the rate of molecular evolution in bacteria. Evolution.

[29] Zuckerkandl E (1976). Evolutionary processes and evolutionary noise at the molecular level. I Functional density in proteins. J Mol Evol.

[30] Sharp PM, Li WH (1987). The rate of synonymous substitution in enterobacterial genes is inversely related to codon usage bias. Mol Biol Evol.

[31] Drummond DA, Raval A, Wilke CO (2006). A single determinant dominates the rate of yeast protein evolution. Mol Biol Evol.

[32] Sharp PM (1991). Determinants of DNA sequence divergence between Escherichia coli and Salmonella typhimurium: codon usage, map position, and concerted evolution. J Mol Evol.

[33] Schattner P, Brooks AN, Lowe TM (2005). The tRNAscan-SE, snoscan and snoGPS web servers for the detection of tRNAs and snoRNAs. Nucleic Acids Res.

[34] Sabi R, Tuller T (2014). Modelling the efficiency of codon-tRNA interactions based on codon usage Bias. DNA Res.

[35] Navon S, Pilpel Y (2011). The role of codon selection in regulation of translation efficiency deduced from synthetic libraries. Genome Biol.

[36] Näsvall SJ, Chen P, Björk GR (2004). The modified wobble nucleoside uridine-5-oxyacetic acid in tRNAPro(cmo5UGG) promotes reading of all four proline codons in vivo. RNA.

[37] Mitchell A, Finch LR (1977). Pathways of nucleotide biosynthesis in Mycoplasma mycoides subsp. mycoides. J Bacteriol.

[38] Bizarro CV, Schuck DC (2007). Purine and pyrimidine nucleotide metabolism in Mollicutes. Genet Mol Biol.

[39] Pollack JD, Myers MA, Dandekar T, Herrmann R (2002). Suspected utility of enzymes with multiple activities in the small genome Mycoplasma species: the replacement of the missing “household” nucleoside diphosphate kinase gene and activity by glycolytic kinases. OMICS.

[40] Galtier N (2003). Gene conversion drives GC content evolution in mammalian histones. Trends Genet.

[41] Williams MV, Pollack JD (1990). A mollicute (mycoplasma) DNA repair enzyme: purification and characterization of uracil-DNA glycosylase. J Bacteriol.

[42] Eyre-Walker AC (1991). An analysis of codon usage in mammals: selection or mutation bias?. J Mol Evol.

[43] Hershberg R, Petrov DA (2010). Evidence that mutation is universally biased towards AT in bacteria. PLoS Genet.

[44] Lynch M, Ackerman MS, Gout JF, Long H, Sung W, Thomas WK, Foster PL (2016). Genetic drift, selection and the evolution of the mutation rate. Nat Rev Genet.

[45] Hildebrand F, Meyer A, Eyre-Walker A (2010). Evidence of selection upon genomic GC-content in bacteria. PLoS Genet.

[46] Cho B-K, Zengler K, Qiu Y, Park YS, Knight EM, Barrett CL (2009). Elucidation of the transcription unit architecture of the Escherichia coli K-12 MG1655 genome. Nat Biotechnol.

[47] Bolstad BM, Irizarry RA, Astrand M, Speed TP (2003). A comparison of normalization methods for high density oligonucleotide array data based on variance and bias. Bioinformatics.

[48] Emms DM, Kelly S (2015). OrthoFinder: solving fundamental biases in whole genome comparisons dramatically improves orthogroup inference accuracy. Genome Biol Genome Biology.

[49] Collingridge PW, Kelly S (2012). MergeAlign: improving multiple sequence alignment performance by dynamic reconstruction of consensus multiple sequence alignments. BMC Bioinformatics.

[50] Wang D, Zhang Y, Zhang Z, Zhu J, Yu J (2010). KaKs_Calculator 2.0: a toolkit incorporating gamma-series methods and sliding window strategies. Genomics, Proteomics Bioinformatics.

[51] Seward EA, Kelly S (2018). Dataset from: Selection-driven cost-efficiency optimisation of transcripts modulates gene evolutionary rate in bacteria [dataset] Zenodo.

