# Supplementary material for: Selection-driven cost-efficiency optimization of transcripts modulates gene evolutionary rate in bacteria
Source: Genome Biol. 2018 Jul 31;19:102. doi: 10.1186/s13059-018-1480-7 (PMC6066932; doi:10.1186/s13059-018-1480-7)
Supplement: Supplementary file 2 — Figure S1, Figure S2 and the CodonMuSe algorithm. (PDF 964 kb) [file 13059_2018_1480_MOESM2_ESM.pdf]

Supplementary Figure 1.

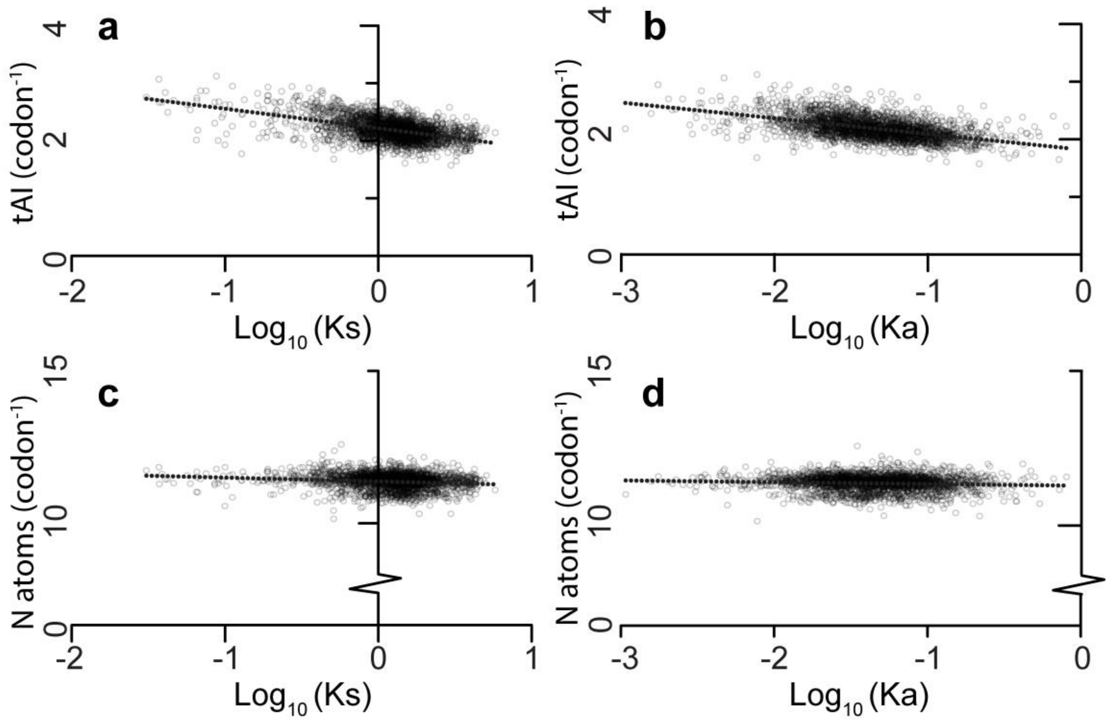

Supplementary Figure 1. Correlation between tAI and codon biosynthetic cost with K_s and K_a for *Escherichia coli* and *Salmonella enterica*.

a) Scatter-plot of $\log_{10}(K_s)$ compared to average tAI per codon per gene (y = -0.3x + 2.2, R^2 = 0.25). **b)** Scatter-plot of $\log_{10}(K_a)$ compared to average tAI per codon per gene (y = -0.3x + 1.8, R^2 = 0.26). **c)** Scatter-plot of $\log_{10}(K_s)$ compared to average cost per codon per gene (y = -0.1x + 11.4, R^2 = 0.02). **d)** Scatter-plot of $\log_{10}(K_a)$ compared to average cost per codon per gene (y = -0.1x + 11.3, R^2 = 0.01).

Supplementary Figure 2.

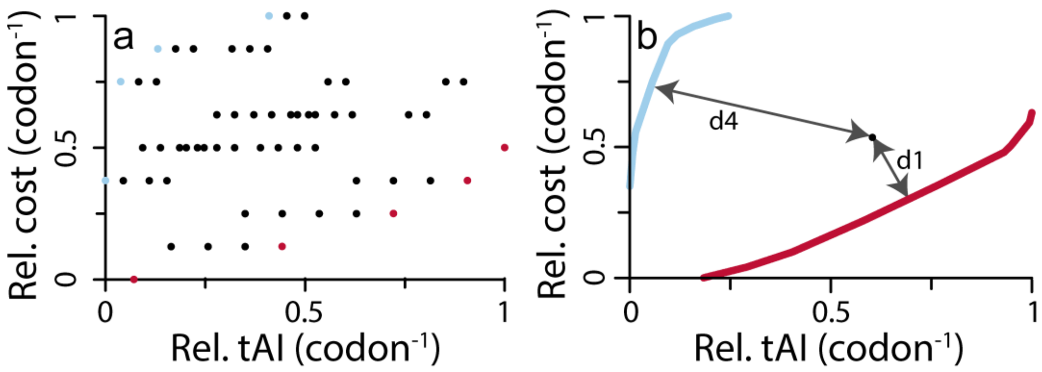

Supplementary Figure 2. Example cost-efficiency Pareto frontier for a short amino acid sequence.

a) Scatter plot of the 64 possible coding sequences encoding the amino acid sequence MTGCD. Red dots indicate coding sequences that are positioned on the best cost-efficiency Pareto frontier (the least expensive, most translationally efficient sequences possible). Blue dots indicate coding sequences that are positioned on the worst cost-efficiency Pareto frontier (the most expensive, least translationally efficient sequences possible). **b)** Evaluating the cost-efficiency optimality of a coding sequence. d1 is the minimum distance between a given coding sequence and the best cost-efficiency Pareto frontier (red) for that amino acid sequence. d4 is the minimum distance of the same gene to the worse cost-efficiency Pareto frontier for that amino acid sequence (blue). The percent optimality of the coding sequence is evaluated as $\left(\frac{d4}{d1+d4}\right) * 100$.

CodonMuSe – an efficient implementation of the SK model for biased synonymous codon use

Codons are often encoded by multiple synonymous codons, however synonymous codon use is biased. Here we present a computationally efficient implementation of the SK model [1] for inferring the contribution of genome-wide GC bias, selection acting on biosynthetic cost and selection acting on translational efficiency to biased patterns of synonymous codon use. The algorithm is called CodonMuSe (***Codon Mu***tation bias and ***Se***lection). CodonMuSe is implemented in python and makes use of the efficient numerical optimisation methods included in scipy. In particular, the algorithm makes use of the Nelder-Mead algorithm and a simplex method to evaluate the numerical minima of the system of equations described below. This algorithm, instructions for use and example files are available under the University of Oxford Academic Use Licence at https://github.com/easeward/CodonMuSe.

The system of equations for the SK model of synonymous codon use

We first consider that selection acts to bias synonymous codon use in proportion to the number of nitrogen atoms contained within each codon. Following previous published work [2–4], we model the selection bias towards codon $\mathcal{C}_i$ as

$$(1) \quad \alpha(\mathcal{C}_i) = e^{S_c N(\mathcal{C}_i)}$$

where $\alpha(\mathcal{C}_i)$ is the selection bias towards codon $\mathcal{C}_i$ that is dependent on codon biosynthetic cost, and where S_c is the strength of selection acting on codon biosynthetic cost. S_c is a composite parameter $(N_g s)$, where s is the selective coefficient and N_g is the effective number of genes at a locus. $N(\mathcal{C}_i)$ is the number of nitrogen atoms in codon $\mathcal{C}_i$.

Secondly, we consider that selection acts to bias synonymous codon use in proportion to the codon's translational efficiency. The tRNA adaptation index (tAI) [5] of a codon is a measure of codon translational efficiency that takes into account both the abundance of iso-accepting tRNAs and wobble-base pairing. Using the equation developed by dos Reis et al

[6] below, and the optimised s_{ij} values for bacteria obtained by Tuller et al [7], tAI values for each codon were evaluated.

$$(2)\ \omega(C_i) = \sum_{j=1}^{n_i} (1 - s_{ij}) tGCN_{ij}$$

Where $\omega(C_i)$is the absolute adaptiveness value for each codon C_i (referred to in the rest of the text as the tAI value), n_i is the number of tRNA isoacceptors that recognise codon C_i, $tGCN_{ij}$ is the gene copy number of the jth tRNA that recognises codon C_i, and s_{ij} is the selective constraint on the efficiency of codon-anticodon coupling.

We model the translational selection bias towards codon $\mathcal{C}_i$ as

$$(3)\ \eta(\mathcal{C}_i) = e^{S_t \omega(\mathcal{C}_i)}$$

where $\eta(\mathcal{C}_i)$ is the translational selection bias towards codon $\mathcal{C}_i$, $\omega(C_i)$ is the tAI value of codon $\mathcal{C}_i$ and S_t is the strength of selection acting on translational efficiency. S_t is a composite parameter $(N_g s)$, where s is the selective coefficient and N_g is the effective number of genes at a locus.

Finally, we consider the effect of genome-wide GC bias on codon bias. GC bias is modelled as

$$(4)\ \delta = \frac{m}{m+1}$$

where δ is the probability that a particular site is A or T given a genome-wide bias towards AT of m as previously described [8]. For example, if there is no bias towards AT or GC m will be 1 and δ will be 0.5 and thus there is an equal likelihood of any site being AT or GC. GC bias towards codon $\mathcal{C}_i$ was evaluated as

$$(5)\ \beta(\mathcal{C}_i) = \delta^{AT}(1 - \delta)^{GC}$$

where AT is the AT content and GC is the GC content of codon $\mathcal{C}_i$. To make the output values of the model easier to interpret, $GC_b$is reported instead of *m* where

$$(6)\ GC_b = -\log(m)$$

such that where $M_b > 0$ indicates a bias towards GC and $M_b < 0$ indicates a bias towards AT. It should be noted here that the GC_b parameter in is a composite parameter that integrates multiple factors contributing to genome-wide GC content bias into the single variable. Such factors include (but are not limited to) the bias of an organism's DNA polymerase, gene conversion, differences in repair efficiency, and selection acting on genome-wide GC content of DNA sequences.

When considering the combined effects of GC_b, S_c and S_t, we model the bias towards codon C_i as the product of equations 2, 4 and 6 as

$$(7)\ \varepsilon(C_i) = \alpha(C_i)\, \beta(C_i)\ \eta(C_i)$$

the genome-wide probability of observing codon C_i for amino acid θ is therefore evaluated as

$$(8)\ \ p(C_i \mid \theta) = \frac{\varepsilon(C_i)}{\sum_{\theta} \varepsilon(C)}$$

That is, the probability of observing codon C_i is the bias towards codon C_i divided by the sum of biases for all codons encoding amino acid θ. Equation 8 satisfies the law of total probability such that a set of synonymous codons have probabilities that sum to one.

Maximum likelihood estimation of model parameters

To infer the values for GC bias and the selection coefficients, the likelihood of observing a sequence or set of sequences for X is evaluated as

$$(9)\ \mathcal{L}(S_c, S_t, GC_b|X) = \prod_{C_i} p(C_i|\theta)^{T_{C_i}}$$

where T_{C_i} is the number of times that codon C_i occurs in the sequence or set of sequences X. The optimal values for S_c, S_t and M_b are those which produce the maximum likelihood and found using the Nelder-Mead algorithm for function optimisation with parameter selection by Akaike information criterion.

Worked example of CodonMuSe

Glycine (G) is encoded by four synonymous codons (GGT, GGC, GGA, GGG). If GC_b, S_c and S_t were all zero (i.e. there was no genome-wide GC bias, no selection acting on biosynthetic cost, and no selection acting on translational efficiency), these codons would be used in equal proportions (25% each). If only M_b were considered, increasing M_b above zero (indicating bias towards GC) would increase the use of GGC and GGG while decreasing the use of GGA and GGT. For example, an GC_b value of 1 would lead to GGC/GGG each being used 37% of the time and GGA/GGT each being used 13% of the time. Similarly an S_c value greater than zero would favour increased use of GGA and GGG (as they each require 15 nitrogen atoms to make) and decreased use of GGC (13 nitrogen atoms) and GGT (12 nitrogen atoms) proportionally. Analogous calculations are performed using the tRNA adaptation index (tAI) of each codon such that where S_t > 0, codons with higher values of tAI are favoured.

Additional testing of CodonMuSe

In order to determine the extent to which CodonMuSe is able to reliably distinguish between the strength of GC bias (GC_b), selection acting on codon cost (S_c) and selection acting on translational efficiency (S_t), additional analysis was conducted.

Firstly, 12 species were selected with correlations between codon cost and translational efficiency that covered the range of correlation values seen across the 1,320 species in the main analysis of this paper (Figure 2e). ie *Kitasatospora setae* has the most negative correlation (-0.04), *Arcobacter butzleri* has the most positive correlation (1.52) and 10 species were chosen at equal intervals between those two extremes. These were *Chlamydia psittaci* (0.25), *Escherichia coli* (0.37), *Arthrobacter sp.* ATCC 21022 (0.46), *Archangium gephyra* (0.54), *Isoptericola dokdonensis* (0.64), *Syntrophobacter fumaroxidans* (0.72), *Marinithermus hydrothermalis* (0.78), *Citromicrobium sp.* JL477 (0.84), *Erythrobacter*

atlanticus (0.89) and *Fervidicola ferrireducens* (0.98). A non-parametric bootstrap analysis was conducted on these 12 species whereby 500 replicates of CodonMuSe were run for each species on a randomly selected set of genes from that species. Genes were selected with replacement, and the number of genes selected was equal to the number of genes in the original genome for that species. Results from this analysis show that the GC_b, S_c and S_t values obtained are normally distributed with a mean value that is the same as the CodonMuSe result run with no bootstrapping (Figure SF1).

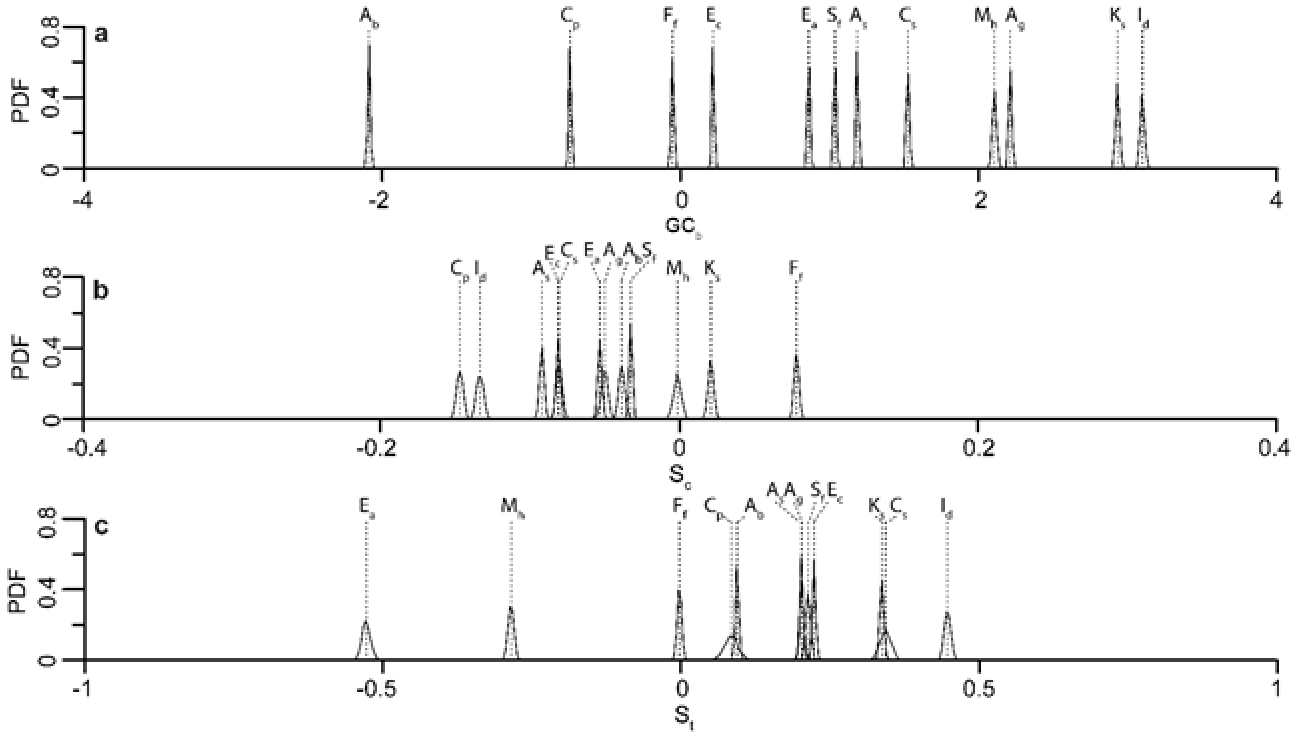

Figure SF1. CodonMuSe estimates for GC_b, S_c and S_t are robust with an average standard deviation < 0.01 and an average 95% confidence interval of ≤ 0.001. A_g = *Archangium gephyra*, A_b = *Arcobacter butzleri*, A_s = *Arthrobacter sp.*, C_p = *Chlamydia psittaci*, C_s = *Citromicrobium sp.*, E_a = *Erythrobacter atlanticus*, E_c = *Escherichia coli*, F_f = *Fervidicola ferrireducens*, I_d = *Isoptericola dokdonensis*, K_s = *Kitasatospora setae* M_h = *Marinithermus hydrothermalis*, S_f = *Syntrophobacter fumaroxidans*. PDF = probability density function. Vertical dotted lines are the CodonMuSe estimated values for that species. a) Average standard deviation for GC_b = 0.007 (range = 0.004 to 0.012). Average 95% confidence interval = 0.001 (range = 0.001 to 0.002). b) Average standard deviation for S_c =

0.002 (range = 0.001 to 0.003). Average 95% confidence interval = 0.000 (range = 0 to 0.001). c) Average standard deviation for S_t = 0.004 (range = 0.001 to 0.011). Average 95% confidence interval = 0.001 (range = 0 to 0.002).

The accuracy of CodonMuSe was further measured by testing it on simulated mRNA sequences generated for 3 different species: *Escherichia coli* (Fig SF2), *Isoptericola dokdonensis* (Fig SF3) and *Citromicrobium sp.* JL477 (Fig SF4). The simulated mRNA sequences were generated from the amino acids sequences of each species as outlined below:

$$(10)\ X = x_0, x_1, x_2, x_3, x_4 \ldots x_n$$

$$(11)\ Y = f(C_{x_0}|M_b, S_c, S_t), f(C_{x_1}|M_b, S_c, S_t), \ldots f(C_{x_n}|M_b, S_c, S_t)$$

$$(12)\ Y = y_0, y, y_2, y_3, y_4 \ldots y_n$$

Where protein sequence X is composed of a string of amino acids from x_0 to x_n. This is converted to mRNA sequence Y using a function that uses the input amino acid (x_n) and the CodonMuSe parameters GC_b, S_c and S_t to simulate codon (y_n) with a probability defined in equation 8. This is implemented using the 'choice' function from numpy.random python module. The range of input values for the simulated sequences was chosen to be at least double the range observed for the 1,320 bacteria in the main analysis of the paper, ie. input values for GC_b used to simulate the sequences ranged from -8 to 8, whereas 95% of the observed GC values for the 1,320 bacterial species fell within the range of -2.4 to 2.4. Input values for S_c used to simulate the sequences ranged from -0.8 to 0.8 and from -2 to 2 for S_t. Since these GC_b, S_c and S_t ranges were sampled at equal intervals and all possible combinations of GC_b, S_c and S_t were tested, there were a total of 9,261 simulated sets of sequences generated for each species (Figures SF2, 3 and 4).

As can be seen in Figures SF2, 3 and 4, simulated GC_b, S_c and S_t values matched fitted values very well within the observed range of GC_b, S_c and S_t seen for the 1,320 bacterial species (denoted by the dashed red lines).

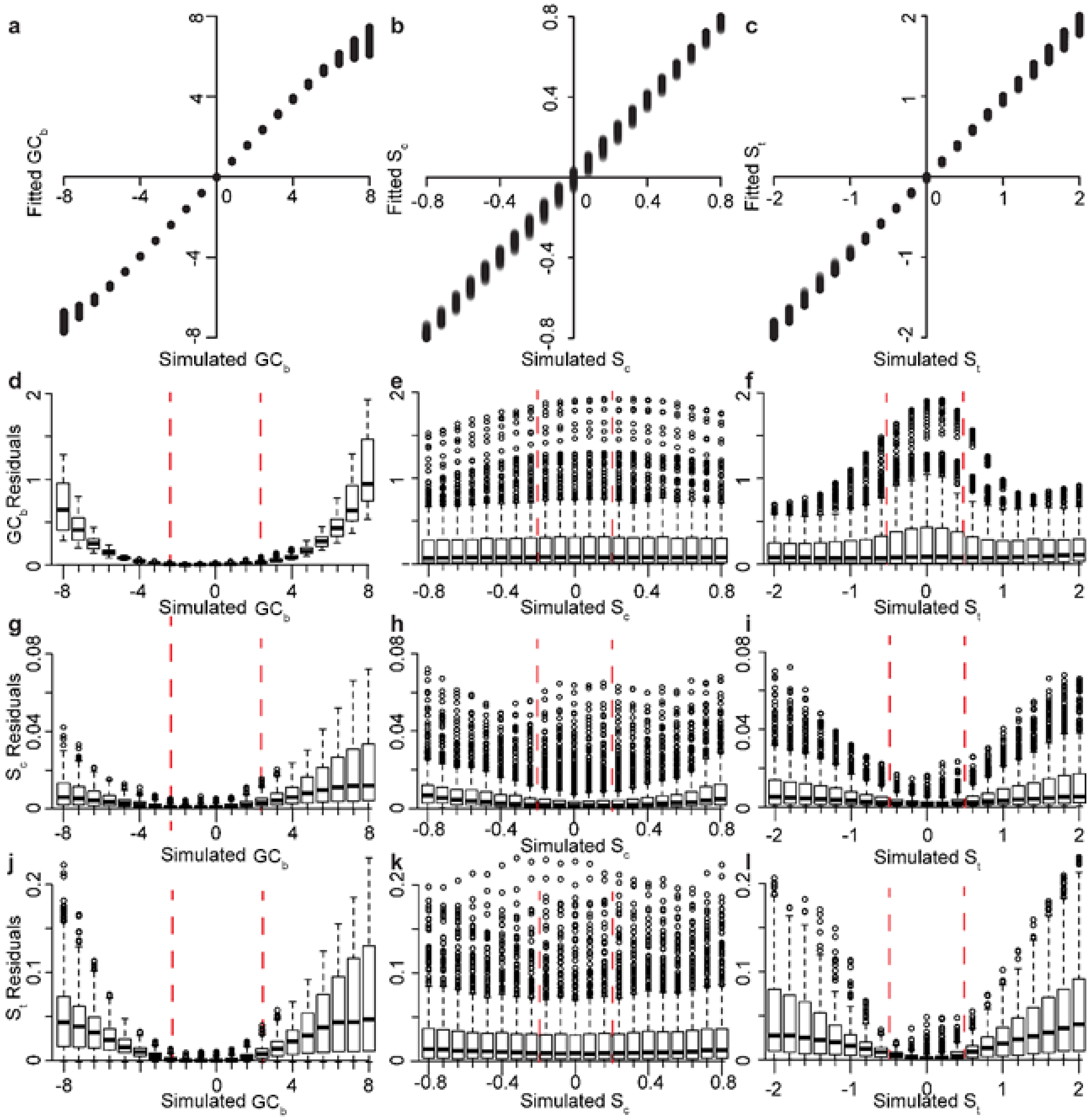

Figure SF2. CodonMuSe is able to accurately reproduce simulated GC_b, S_c and S_t values for *Escherichia coli.* Scatter-plots of simulated verses fitted values for a) GC_b, b) S_c and c) S_t. Box-plots showing M_b residuals for simulated d) GC_b, e) S_c and f) S_t. Box-plots showing S_c residuals for simulated g) GC_b, h) S_c and i) S_t. Box-plots showing S_t residuals for simulated j) GC_b, k) S_c and l) S_t. Red lines denote the values of GC_b, S_c and S_t which contain 95% of the 1,320 bacterial species in the main analysis in this paper.

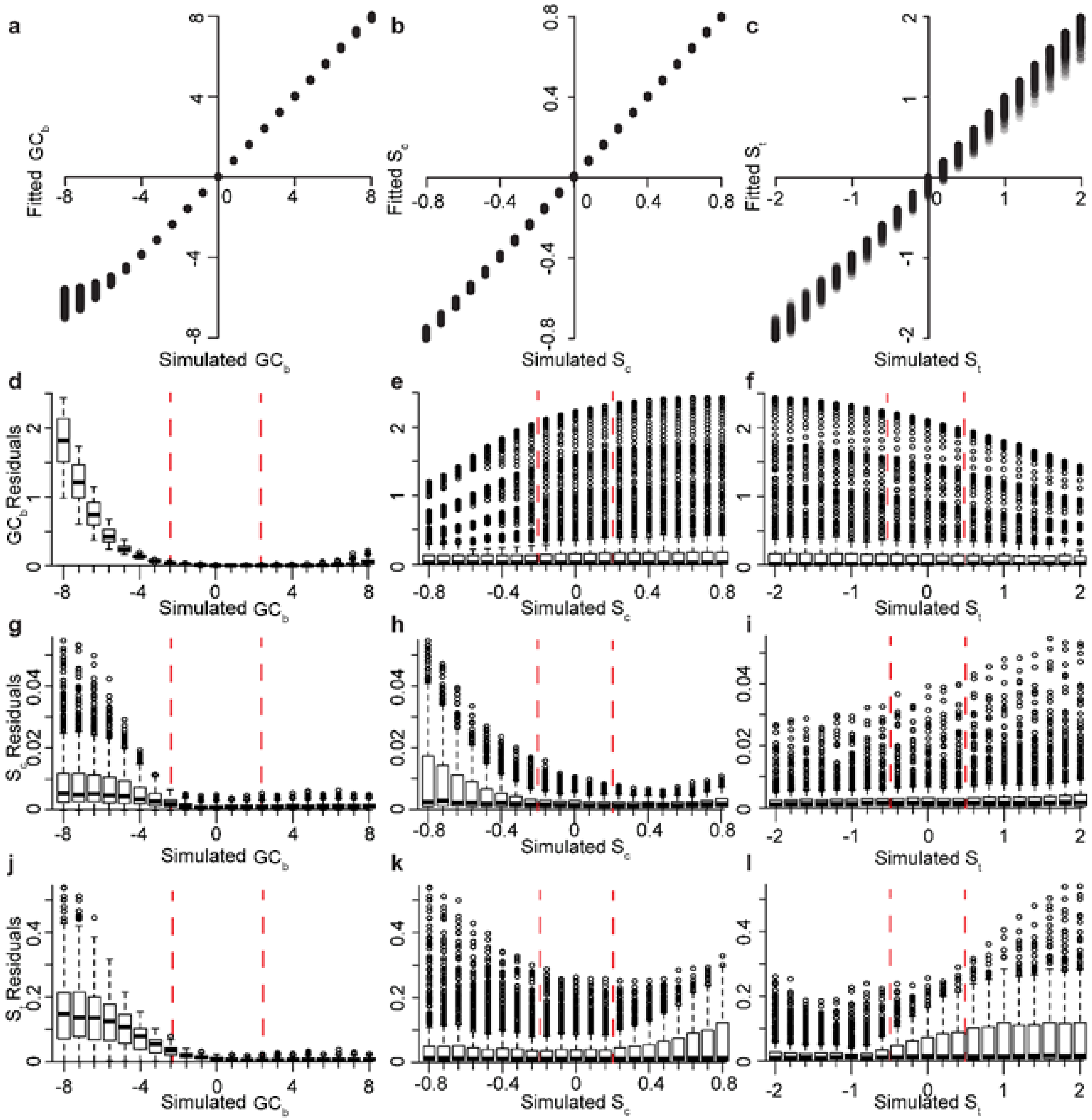

Figure SF3. CodonMuSe is able to accurately reproduce simulated GC_b, S_c and S_t values for *Isoptericola dokdonensis.* Scatter-plots of simulated verses fitted values for a) GC_b, b) S_c and c) S_t. Box-plots showing GC_b residuals for simulated d) GC_b, e) S_c and f) S_t. Box-plots showing S_c residuals for simulated g) GC_b, h) S_c and i) S_t. Box-plots showing S_t residuals for simulated j) GC_b, k) S_c and l) S_t. Red lines denote the values of GC_b, S_c and S_t which contain 95% of the 1,320 bacterial species in the main analysis in this paper.

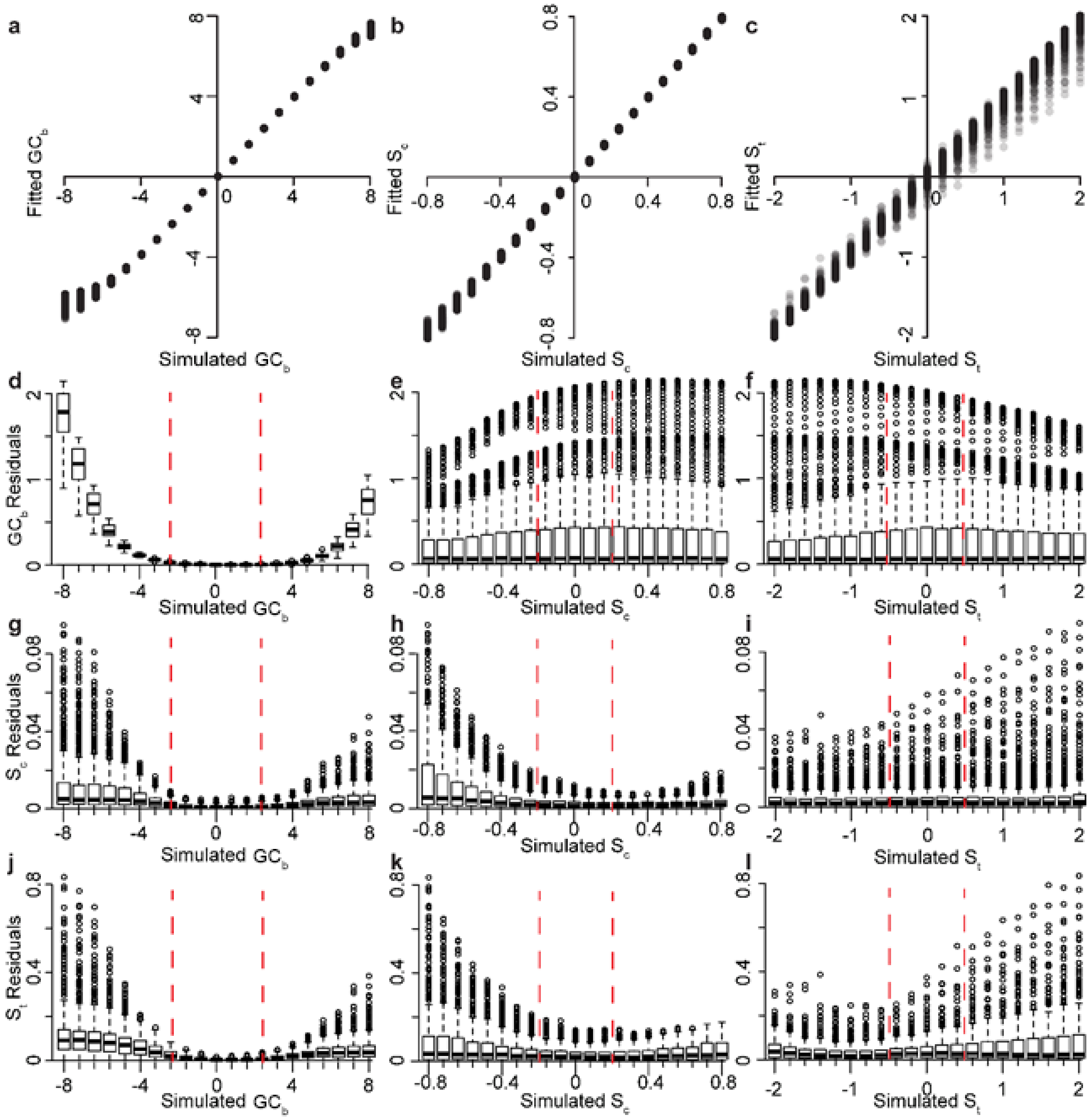

Figure SF4. CodonMuSe is able to accurately reproduce simulated GC_b, S_c and S_t values for *Citromicrobium sp.* JL477. Scatter-plots of simulated verses fitted values for a) GC_b, b) S_c and c) S_t. Box-plots showing M_b residuals for simulated d) GC_b, e) S_c and f) S_t. Box-plots showing S_c residuals for simulated g) GC_b, h) S_c and i) S_t. Box-plots showing S_t residuals for simulated j) GC_b, k) S_c and l) S_t. Red lines denote the values of GC_b, S_c and S_t which contain 95% of the 1,320 bacterial species in the main analysis in this paper.

References

1. Seward EA, Kelly S. Dietary nitrogen alters codon bias and genome composition in parasitic microorganisms. Genome Biol. 2016;17:1–15.

2. Li WH. Models of nearly neutral mutations with particular implications for nonrandom usage of synonymous codons. J. Mol. Evol. 1987;24:337–45.

3. Shields DC. Switches in species-specific codon preferences: The influence of mutation biases. J. Mol. Evol. 1990;31:71–80.

4. Bulmer M. The selection-mutation-drift theory of synonymous codon usage. Genetics. 1991;129:897–907.

5. dos Reis M, Wernisch L, Savva R. Unexpected correlations between gene expression and codon usage bias from microarray data for the whole Escherichia coli K-12 genome. Nucleic Acids Res. 2003;31:6976–85.

6. dos Reis M, Savva R, Wernisch L. Solving the riddle of codon usage preferences: A test for translational selection. Nucleic Acids Res. 2004;32:5036–44.

7. Sabi R, Tuller T. Modelling the Efficiency of Codon – tRNA Interactions Based on Codon Usage Bias. DNA Res. 2014;21:511–25.

8. Lynch M. Chapter 6, The Origins of Genome Architecture. First Edit. Sunderland, MA: Sinauer Associates, Inc. Publishers.; 2007.
